# Neuroprotective Effect of SGLT2 Inhibitors

**DOI:** 10.3390/molecules26237213

**Published:** 2021-11-28

**Authors:** Agnieszka Pawlos, Marlena Broncel, Ewelina Woźniak, Paulina Gorzelak-Pabiś

**Affiliations:** Laboratory of Tissue Immunopharmacology, Department of Internal Diseases and Clinical Pharmacology, Medical University of Lodz, Kniaziewicza 1/5, 91-347 Lodz, Poland; agnieszka.sanetra@stud.umed.lodz.pl (A.P.); ewelina.wozniak@umed.lodz.pl (E.W.); paulina.gorzelak-pabis@umed.lodz.pl (P.G.-P.)

**Keywords:** SGLT2i, sodium-glucose cotransporter 2 inhibitors, neuroprotection, atheroprotection, mTOR, type 2 diabetes mellitus, cognitive impairment, inflammation, oxidative stress

## Abstract

Patients with diabetes are at higher risk of cardiovascular diseases and cognitive impairment. SGLT2 inhibitors (Empagliflozin, Canagliflozin, Dapagliflozin, Ertugliflozin, Sotagliflozin) are newer hypoglycemic agents with many pleiotropic effects. In this review, we discuss their neuroprotective potential. SGLT2 inhibitors (SGLT2i) are lipid-soluble and reach the brain/serum ratio from 0.3 to 0.5. SGLT receptors are present in the central nervous system (CNS). Flozins are not fully SGLT2-selective and have an affinity for the SGLT1 receptor, which is associated with protection against ischemia/reperfusion brain damage. SGLT2i show an anti-inflammatory and anti-atherosclerotic effect, including reduction of proinflammatory cytokines, M2 macrophage polarization, JAK2/STAT1 and NLRP3 inflammasome inhibition, as well as cIMT regression. They also mitigate oxidative stress. SGLT2i improve endothelial function, prevent remodeling and exert a protective effect on the neurovascular unit, blood-brain barrier, pericytes, astrocytes, microglia, and oligodendrocytes. Flozins are also able to inhibit AChE, which contributes to cognitive improvement. Empagliflozin significantly increases the level of cerebral BDNF, which modulates neurotransmission and ensures growth, survival, and plasticity of neurons. Moreover, they may be able to restore the circadian rhythm of mTOR activation, which is quite a novel finding in the field of research on metabolic diseases and cognitive impairment. SGLT2i have a great potential to protect against atherosclerosis and cognitive impairment in patients with type 2 diabetes mellitus.

## 1. Introduction

Type 2 diabetes mellitus (T2DM) is a chronic metabolic disease causing a variety of complications, including atherosclerosis which is associated with increased cardiovascular risk contributing to reduced life expectancy [1]. Additionally, atherosclerosis is an important factor leading to cognitive impairment in the elderly via several mechanisms such as ischemia and a direct molecular link [2,3]. Diabetes mellitus type 2 accelerates the development of atherosclerosis, and patients with T2DM are at a two to four times higher risk of developing vascular diseases than non-diabetics [4]. There is a lot of evidence that proves that diabetic patients are at an increased risk of developing cognitive impairment. Glucose metabolism is also impaired in Alzheimer’s disease, as it is sometimes called ‘Type 3 diabetes’ or ‘diabetes of the brain’ [5]. According to a meta-analysis performed by Zhang J. et al., patients with diabetes mellitus type 2 have a 53% higher relative risk of Alzheimer’s disease than non-diabetic individuals (RR 1.53, 95% CI: 1.42–1.63) [6]. Among diabetics, the presence of micro- and macrovascular complications increases the risk of cognitive decline even further, suggesting that vascular mechanisms, including atherosclerosis, are important players [7]. As diabetic patients with atherosclerosis are especially vulnerable to cognitive impairment, it is necessary to search for drugs that could ensure T2DM control, reduce cardiovascular risk and improve cognitive functions. 

SGLT2 inhibitors are newer hypoglycemic drugs that have revolutionized the clinical approach to T2DM management. Their main mechanism of action is inhibiting SGLT2 receptors in the proximal tubules of the kidneys and thus lowering blood glucose levels by blocking its reabsorption from the urine [8]. As it has been proved by large double-blind clinical trials, Empagliflozin not only decreases HbA1c in diabetic patients but also improves their life expectancy by reducing cardiovascular mortality [9]. Canagliflozin, Dapagliflozin, Sotagliflozin significantly decrease the composed primary end-point, including cardiovascular mortality and other cardiovascular outcomes [10,11,12]. Ertugliflozin showed non-inferiority vs. placebo in reducing cardiovascular mortality and other cardiovascular outcomes [13]. The exact mechanism has not been fully established yet, and SGLT2 inhibitors show many additional beneficial effects which contribute to their wider use, even in non-diabetic patients [14]. There is growing evidence that SGLT2 inhibitors have a neuroprotective potential, as in a murine mixed model of diabetes mellitus and Alzheimer’s disease, empagliflozin improved both cerebral microvascular and cognitive impairment [15]. There is no available data on the adverse effects flozins may exert on the Central Nervous System. The most commonly known side effects are genitourinary infections; however, rare but more serious effects also may occur, like euglycemic ketoacidosis [16]. In this review, we are focusing on SGLT2 inhibitors’ potential to improve the impaired cognitive functions of diabetic patients with atherosclerosis.

## 2. Neurological Potential of SGLT2 Inhibitors

**Figure 1:**
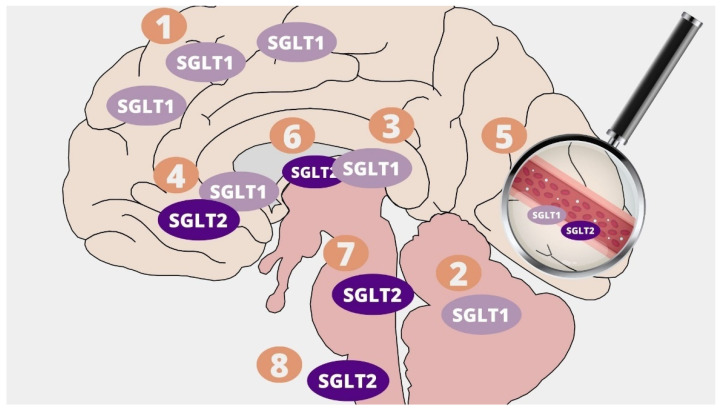
Distribution of SGLT1 and SGLT2 receptors in the Central Nervous System: 1. Pyramidal cells of brain cortex; 2. Purkinje cerebellum cells; 3. Hippocampus pyramidal and granular cells; 4. Hypothalamus; 5. Microvessels; 6. Amygdala; 7. Periaqueductal grey; 8. Dorsomedial medulla—nucleus of the solitary tract (NTS).

SGLT2 inhibitors are lipid-soluble and cross the blood-brain barrier reaching the brain-to-serum ratio of the areas under the curves from 0.3 (Canagliflozin and Dapagliflozin) up to 0.5 (Empagliflozin) [17]. They have the ability to directly affect their target, since SGLT1 and SGLT2 co-receptors are expressed in the human central nervous system and play an important role in maintaining glucose homeostasis. SGLTs are responsible for the transport of glucose, galactose and sodium ions against the concentration gradients [18]. SGLT1 transports two Na+ ions with one D-glucose molecule and SGLT2 one sodium ion with one D-glucose [19]. They may be found in many areas of the central nervous system in several isoforms. SGLT1 inhibitors are present in the pyramidal cells of the brain cortex, Purkinje cerebellum cells, hippocampus pyramidal, and granular cells [20]. They were also detectable in glial cells in the ventromedial hypothalamus [21]. Brain expression of SGLT2 is lower than SGLT1, and it occurs mainly in the microvessels of the blood-brain barrier, but also in the amygdala, hypothalamus, periaqueductal gray (PAG), and in the dorsomedial medulla – the nucleus of the solitary tract (NTS) [22,23]. The presence of SGLT1/SGLT2 was also described in the abluminal membrane of the capillary endothelium [21]. Interestingly, the brain locations where SGLTs are present have been proven to be responsible for learning processes, food intake, energy and glucose homeostasis, and central cardiovascular and autonomic regulation [23,24]. The location of SGLT1 and SGLT2 receptors in the CNS is presented in Figure 1. It is possible that SGLT2 receptors also exert a cardioprotective effect through central mechanisms by directly influencing cardiovascular regulation and autonomic pathways, including the paraventricular nucleus of the hypothalamus, the nucleus of the solitary tract, and the periaqueductal gray [23]. An immunoblotting study of post-mortem human brain tissue showed a significant increase in SGLT1 and SGLT2 expression following brain injury [25]. Results of a study performed in a murine model suggest that, after a brain injury, SGLT1 blockage may bring beneficial effects with regard to the area of the brain lesions, the volume of damaged tissue, edema, and motoric disability [26].

SGLT2 inhibitors are not fully selective for SGLT2 co-receptors, and they also affect SGLT1 to various extents (Table 1). Sotagliflozin has the most affinity to SGLT1 receptors. It is even called a “dual SGLT1/SGLT2 inhibitor”, however, it is the newest Flozin, and it is not yet used in diabetic patients on a large scale [12]. Among commonly used SGLT2 inhibitors, Canagliflozin has the greatest potential for inhibiting SGLT1 receptors. In contrast, Empagliflozin and Ertugliflozin are the most selective for SGLT2 and have the lowest potential for interaction with SGLT1 [27]. Therefore, theoretically, to obtain the neuroprotective effect associated with SGLT1 inhibition in diabetic patients, Sotagliflozin and Canagliflozin should be preferred over Dapagliflozin, Empagliflozin, and Ertugliflozin.

In the central nervous system, there is also a place for selective SGLT2 inhibitors since, based on the results obtained by Erdogan MA. et al., Dapagliflozin significantly reduces seizure activity, both at the electrophysiological and clinical level, in a rat model of epilepsy [32]. It may be associated with a similar effect of glucose fasting-like metabolic switch as the one observed in ketogenic diets, which in some circumstances also improve brain seizure activity [54]. There is no clinical data comparing the efficacy of ketogenic diets and dapagliflozin therapy on brain epileptic activity; however, adhering to a ketogenic diet is difficult and has to be closely monitored. On the contrary, dapagliflozin is a safe drug widely used in diabetic patients. Interestingly, cognitive impairment shares the same risk factors as epilepsy and atherosclerosis, and commonly used anti-epileptic drugs such as phenytoin, carbamazepine, valproic acid are associated with increased cardiovascular risk [55]. Dapagliflozin may be a preferable flozin in diabetic patients with epilepsy as it has an anti-epileptic potential. Moreover, it significantly reduces cardiovascular risk and thus may prevent cognitive decline.

There is in vivo evidence for the expression of SGLT2 protein in choroid plexus epithelial cells and ependymal cells of the human brain [56]. This is crucial information indicating that SGLT2 may have an influence on the composition of the cerebrospinal fluid (CSF), whose role in the pathology of neurodegenerative disorders provides a new direction for research and requires further investigation [57].

There is growing evidence that apart from direct mechanisms of SGLT2 inhibitors in the central nervous system, they also exert a beneficial pleiotropic effect. In silico studies indicate that flozins have the molecular ability to inhibit acetylcholinesterase. Canagliflozin was even called a ‘dual inhibitor of SGLT2 and AChE’ as its estimated inhibition constant *K*_i_ (i.e., the concentration required to produce half-maximum inhibition) against AChE was 0.12859 µM [58]. It is clinically relevant as patients taking canagliflozin reach a serum drug concentration of 10µM, and the brain/serum ratio of canagliflozin is 0.3. Therefore, the amount of canagliflozin penetrating the brain (3 µM) is enough to inhibit AChE [17,59]. As for other SGLT2i, the *K*_i_ for inhibiting AChE is 0.177 µM for empagliflozin and 25.02 µM for dapagliflozin, and brain concentrations are 0.5 µM and 0.3 µM, respectively, so out of those two, only in the case of empagliflozin, brain concentration is enough to inhibit AChE (Table 1) [17,30,60]. Patients with Alzheimer’s disease have a reduced amount of acetylcholine neurotransmitters in the brain, and acetylcholinesterase inhibitors including donepezil, rivastigmine, galantamine are commonly used to increase the acetylcholine level and improve cognition [61]. In a rat model of cognitive impairment induced by scopolamine, canagliflozin, similarly to galantamine, decreased AChE activity, increased acetylcholine M1 receptor (M1 mAChR) and monoamines levels. It also improved cognitive functions in the Y maze task and water maze task [62]. Canagliflozin has the greatest potential of inhibiting AChE and may be a preferable solution in patients with T2DM who would also benefit from the inhibition of acetylcholinesterase.

Another promising effect SGLT2i exert on the central nervous system was described by Lin B. et al., for empagliflozin, which significantly increased cerebral BDNF (Brain-derived neurotrophic factor) levels in db/db mice (Table 1). Moreover, this effect was accompanied by improvement in cognitive functions [31]. BDNF takes part in the growth, survival, and plasticity of neurons as well as in the modulation of neurotransmission. It is an important factor for the processes of learning and memorizing [63]. Interestingly, a significant decline of BDNF was observed in patients with T2DM, and it was associated with cognitive impairment, which was not observed in non-diabetic controls [64]. Surprisingly, BDNF is crucial not only for the central nervous system but also for atherosclerosis. Patients with DMAS (Diabetes Mellitus Accelerated Atherosclerosis) had a lower expression of BDNF, and it was negatively correlated with inflammation. In the same study, supplementation of BDNF in mice significantly reduced atherosclerotic lesions [65]. The anti-inflammatory properties of BDNF are probably associated with promoting M2 macrophages polarization via STAT3 [65]. SGLT2i may thus bring benefits to diabetic patients with atherosclerosis by preventing cognitive impairment associated with low levels of BDNF.

## 3. Atherosclerosis, Cognitive Impairment, and SGLT2i

The presence of cholesterol-rich plaques in the walls of large cerebral arteries is defined as Cerebral Atherosclerosis (CA). In previous studies, atherosclerotic lesions in intra- and extra-cranial arteries were associated with cognitive impairment and even dementia [2,66,67]. According to results obtained by Dearborn JL. et al., atherosclerotic plaques in the anterior cerebral artery occurred independently of vascular risk factors associated with increased prevalence of dementia (RPR 3.81 95%CI [1.57–9.23] *p* = 0.003) in elderly patients [2]. On the other hand, atherosclerosis in the posterior cerebral artery increased the risk of Mild Cognitive Impairment (MCI) (RPR 1.44 95% CI [1.04–1.98] *p* = 0.027) [2]. As demonstrated by another study, dementia occurred more frequently in patients with atherosclerotic calcifications in intra and extra-cranial arteries as opposed to coronary vessels [66]. Cerebral atherosclerosis and dementia are related to each other; however, the exact mechanism remains unknown. In reference to the proteomic sequencing of the dorsolateral prefrontal cortex of 438 humans performed by Wingo A.P. et al., CA was associated with reduced synaptic function, excess myelination, and axonal injury independently of ischemia [67]. Preventing atherosclerosis would contribute to the improvement in cognitive functions in elderly people. Results obtained by Sabia S. et al. show that cardiovascular health at the age of 50 years is crucial for further development of cognitive impairment [68]. As mentioned before, SGLT2i significantly reduce cardiovascular risk. They exert a pleiotropic anti-atherosclerotic effect by reducing vascular inflammation, oxidative stress and improving endothelial dysfunction [69]. In a previous study including diabetic patients, a three-month treatment with empagliflozin resulted in a significant regression of complex intima media thickness (cIMT) by 7.9%; *p* < 0.0001. Interestingly, this effect was significant just after one month of empagliflozin therapy [34]. CIMT is a relevant marker of early atherosclerosis, and it is often measured in the carotid arteries [70]. According to Feinkohl I. et al., cIMT is also a significant predictor of cognitive decline in patients with T2DM [71]. Future studies should evaluate the clinical relevance of the ability of SGLT2 to reduce atherosclerotic lesions and thus the impact on cognitive functions. 

## 4. Inflammation

The inflammatory process in the central nervous system also referred to as neuroinflammation, is associated with a lot of pathologies, including cognitive dysfunction. In a study conducted by Suridjan I. et al., and including patients with Alzheimer’s disease, the presence of neuroinflammation detected by [18F]-FEPPA was positively correlated with the level of cognitive decline [72]. There is growing evidence that the presence of inflammation outside the central nervous system (systemic inflammation) can also contribute to a decline in cognitive functions [73]. According to Walker K. et al., elevated inflammatory markers in middle adulthood resulted in significant cognitive decline after 20 years [74]. The neurovascular unit (NVU), which is composed of endothelial cells lining brain microvessels as well as neurons, microglia, astrocytes, and pericytes, mediates homeostasis by regulating traffic between blood and the neural environment. Systemic inflammation is associated with circulating proinflammatory cytokines, which impair the endothelium of brain microvessels, increase the permeability of the blood-brain barrier and change the phenotype of astrocytes and microglia into pro-inflammatory ones [75]. M1 activated microglia impair NVU by secreting proinflammatory cytokines, including TNF-α, IL-1β, IL-6, IL-18, which contribute to neurodegeneration by breaking neurotransmitters into bioactive metabolites, tau hyperphosphorylation, β-amyloid oligomerization, and complement activation [76,77]. In a mouse model of T2DM, empagliflozin had a protective effect, involving remodeling prevention on the neurovascular unit, the blood-brain barrier, pericytes, astrocytes, microglia, and oligodendrocytes [49]. The inflammatory process is also a key driver of atherosclerosis. In the CANTOS study, inhibition of interleukin-1β with canakinumab significantly reduced cardiovascular risk independently of lipid levels [78]. Proinflammatory cytokines including TNF-α, IL-1β, IL-6 are also a mediator of atherosclerosis since they activate endothelial cells, attract monocytes, and facilitate their adhesion by up-regulating MCP-1, ICAM, VCAM [79]. There is abundant evidence from animal studies showing that SGLT2 inhibitors slow down the progression of atherosclerosis and exert an anti-inflammatory effect by reducing the expression of proinflammatory cytokines, including TNF-α, IL-1β, IL-6, MCP-1, ICAM, VCAM [69]. In humans, the serum level of IL-6 dropped by 26.6% (*p*  =  0.010) after 2 years of canagliflozin treatment [35].

NLRP3 (NOD-, LRR- and pyrin domain- containing protein 3) inflammasome activation is one of the key molecular pathways mediating inflammation as it leads to the release of IL-1β and IL-18 cytokines. It is a crucial element of the innate immune system activated not only by microbial infection or cellular damage but also by chronic inflammatory diseases, including atherosclerosis and Alzheimer’s disease [80]. NLRP3 is an important mechanism that drives inflammation in atherosclerosis since activation of this pathway in arterial walls by lipoproteins triggers inflammatory response [81]. In a mouse model of atherosclerosis, the inhibition of the NLRP3 inflammasome by MCC950 resulted in a significant reduction in atherosclerotic lesions [82]. In Alzheimer’s disease, NLRP3 inflammasome links systemic inflammation with neuroinflammation and impairs the removal of amyloid-beta by the microglia [83]. This effect may be clinically significant, as, in another study, the inhibition of NLRP3 by OLT1177 significantly improved cognitive impairment in a mouse model of Alzheimer’s disease [84]. SGLT2 inhibitors may improve atherosclerosis and cognitive dysfunction via NLRP3 inflammasome inhibition (Table 1). As proven by Kim S. et al., in the ex vivo study in diabetic patients, empagliflozin significantly attenuated the inflammasome activity after 30 days of treatment [40].

**Figure 2:**
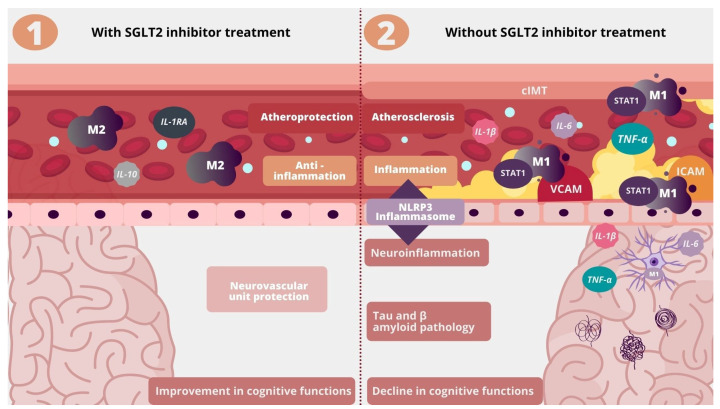
Influence of SGLT2 inhibitors on inflammation, atherosclerosis, and neuroinflammation. IL-1RA—Interleukin 1 Receptor Agonist, cIMT—carotid intima-media thickness, STAT1—Signal transducer and activator of transcription 1, VCAM—Vascular Cell Adhesion Molecule; ICAM-Intracellular Adhesion Molecule.

Macrophages play a central role in atherosclerosis due to their foam cell formation in the vascular lesions. They are also an important factor in the pathology of cognitive impairment associated with Alzheimer’s disease as their infiltration is increased in patients with AD being most abundant in the brain regions rich in Aβ plaques [85]. Macrophages are immune cells responsible for mediating chronic low-grade inflammation and residual cardiovascular risk, which remains after lipid reduction. They are characterized by an ability to change in response to the environment. There are two immunological types of macrophages, i.e., M1 and M2 macrophages. M1 proinflammatory macrophages secrete 1β, IL-6, and TNF-α, maintain a chronic inflammatory state, and promote atherogenesis. On the contrary, M2 macrophages have an anti-inflammatory and atheroprotective profile by secreting IL-1 receptor agonist, IL-10, and collagen [86]. SGLT2 inhibitors have been proven to strongly promote macrophage polarization towards M2 and thus alleviate inflammation and atherosclerosis (Table 1) [42]. In the central nervous system, M1 polarization of glial cells was associated with neurodegeneration [73]. M1 polarized macrophages activate STAT-1, which is a proinflammatory transcription factor [87]. It can also be involved in cognitive impairment in Alzheimer’s disease since it is activated by intracellular Tau accumulation. In a murine model, depletion of STAT-1 activation significantly reduced synaptic dysfunction and cognitive impairment associated with Tau accumulation [88]. Empagliflozin was proven to mitigate inflammation by downregulation of the JAK2/STAT1 pathway in macrophages [89]. Macrophages take part in cognitive impairment as perivascular macrophages (PVM) are the source of vascular oxidative stress by producing a large amount of free radicals near the neurovascular unit [90]. They also affect the permeability of the blood-brain barrier [91]. In previous studies, depletion of perivascular macrophages prevented short-term memory impairment in a murine model [92]. SGLT2 inhibitors may possibly attenuate atherosclerosis and cognitive impairment via macrophages by promoting M2 polarization and downregulating STAT-1 (Figure 2).

## 5. Oxidative Stress and Mitochondrial Dysfunction

A chronic inflammatory state also contributes to oxidative damage as it causes the release of reactive oxygen species (ROS) [93]. The overproduction of ROS or decrease in the anti-oxidant defense results in oxidative stress, which is a significant contributor to vascular diseases, including atherosclerosis, as it causes endothelial dysfunction, promotes remodeling and further enhances inflammation [94]. Oxidative stress is also associated with Aβ- or tau -induced neurotoxicity since it facilitates their aggregation, phosphorylation, and polymerization. These processes contribute to neurodegeneration which results in impaired synaptic plasticity, neuroinflammation, neurotransmitter imbalance, neuronal and synaptic loss leading to cognitive impairment [95]. In the previous study, the increased level of oxidative stress was associated with cognitive decline in a healthy population [96]. Interestingly, SGLT2 inhibitors were proven to ameliorate oxidative stress not only by maintaining a normal glucose level but also by reducing the generation of free radicals (Table 1) [97]. In patients with T2DM, empagliflozin significantly enhanced leukocyte expression of antioxidative enzymes including glutathione s-reductase and catalase and simultaneously reduced pro-oxidative myeloperoxidase after four months of treatment [47].

Mitochondrial function is crucial for maintaining neuronal homeostasis, as neurons are vulnerable to bioenergetic changes. Mitochondrial dysfunction plays an important role in the pathogenesis of neurodegenerative diseases; there is even a ”Mitochondrial Cascade hypothesis” in Alzheimer’s Disease pathology [98]. In a murine model, depletion of AIF (apoptosis-inducing factor), which is a mitochondrial protein taking part in apoptosis and electron transport chain, was associated with serious disturbances in hippocampal-dependant spatial learning and memory [99]. In a rat model, taking dapagliflozin was associated with significant improvement in brain mitochondrial function, including decreased ROS production, mitochondrial swelling, and mitochondrial membrane depolarization [100]. The existing evidence supports the concept that SGLT2 may improve atherosclerosis and cognitive impairment by reduction in oxidative stress and improvement in mitochondrial dysfunction.

## 6. mTOR Signaling

**Figure 3:**
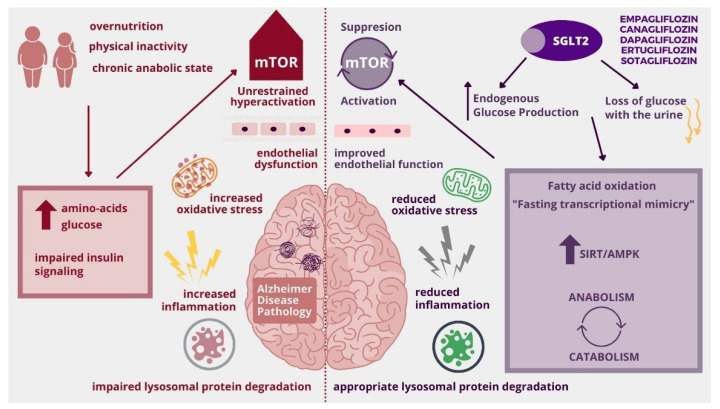
Influence of SGLT2 inhibitors on unrestrained activation of mTOR (mechanistic/mammalian target of rapamycin). AMPK-AMP-activated protein kinase, SIRT-Sirtuin.

mTOR (mechanistic/mammalian target of rapamycin) is a novel, promising molecular pathway linking metabolic diseases and cognitive impairment. It is a crucial cellular coordinator of systemic energy status and local nutrients. Chronic up-regulation of mTOR is present in an anabolic state (increased levels of glucose, amino acids, growth factors) associated with over-nutrition and lack of physical activity [101]. Continuous activation of mTOR causes endothelial cell dysfunction, which is not only a key point of atherosclerosis but also contributes to interruption in the blood-brain barrier [102]. Unrestrained mTOR up-regulation has also been linked to tau and amyloid β hyperphosphorylation and aggregation in Alzheimer’s disease [103]. Moreover, chronic mTOR activation impairs lysosomal protein degradation, which supports the “Endo-Lysosomal Dysfunction” hypothesis of Alzheimer’s Disease [104]. It is believed that restoring the circadian rhythm of mTOR activation would be beneficial in metabolic diseases and cognitive impairment. This effect can be achieved by increasing physical activity, reducing calories intake, or intermittent fasting. All the abovementioned interventions require the patient’s determination and are difficult to obtain in real-life clinical practice. SGLT2 inhibitors are able to mimic those states by promoting catabolism and restoring mTOR cycling, thus decreasing cognitive impairment associated with metabolic diseases [105]. An interesting SGLT2i effect was noticed by Esterline R. et al.; SGLT2 inhibitors cause loss of glucose with urine, but simultaneously they activate glycogenolysis and gluconeogenesis and thus increase fasting endogenous glucose production, which occurs particularly at night. This effect contributes to switching metabolism from anabolic to catabolic depending not on glucose and insulin but on fatty acid oxidation and leads to a decrease in mTOR fuel: blood insulin and amino-acids. Nocturnal mTOR suppression is followed by daily activation, and this state allows maintaining mitochondrial and lysosomal homeostasis (Figure 3) [104]. Additionally, according to Packer M, SGLT2 inhibitors cause transcriptional changes in cells that occur during starvation, which is called “state of fasting mimicry” and include SIRT/AMPK activation and Akt/mTOR suppression (Figure 3). Moreover, taking flozins causes changes similar to an ischemic state, including HIF-2α activation which stimulates erythropoiesis, and patients with higher erythrocyte count benefited most from SGLT2i therapy. Interestingly those effects occurred also in cells, which do not express SGLT [106]. There is a lot of evidence that SGLT2 inhibitors are able to suppress mTOR (Table 1). Flozins, by restoring the circadian rhythm of mTOR activity, seem to bring benefits in patients with Alzheimer’s Disease according to “Type 3 Diabetes Hypothesis”, “Mitochondrial Cascade Hypothesis” and “Endo-Lysosomal Dysfunction Hypothesis” [105].

## 7. Cerebrovascular Dysfunction

Cerebrovascular dysfunction is a pathological condition of the brain related to vascular pathology. A hyperglycemic state impairs the microvascular structure of the brain causing neurovascular remodeling, including loss of endothelial integrity, basement membrane thickening, loss of myelin and neurons, astrocytes and pericytes disturbance [107]. Such ultrastructural changes are associated with cognitive decline [108]. In a mouse model of T2DM, empagliflozin exerted a neuroprotective effect on neurovascular remodeling [49].

**Figure 4:**
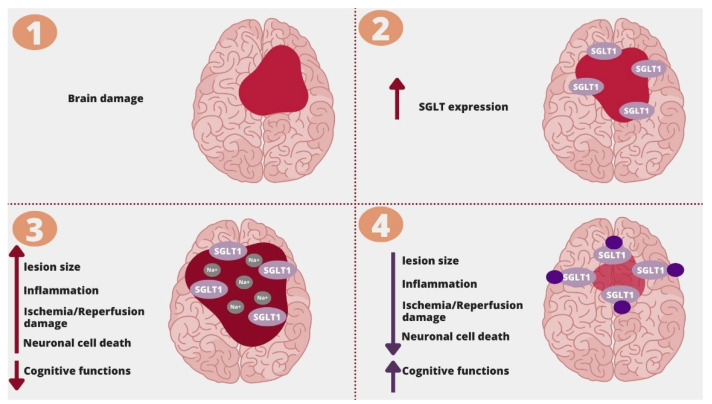
SGLT1 inhibition and ischemic brain damage. 1. Brain damage; 2. In the area of brain damage, there is an increase in the expression of SGLT1; 3. Sodium influx through SGLT1 receptors is associated with increased ischemia/reperfusion damage, lesion size, edema, inflammation, neuronal cell death, and decline in cognitive functions; 4. SGLT receptor blockage/knockdown was associated with improvement in damages caused by ischemia and ischemia/reperfusion damage.

Cerebrovascular dysfunction is mainly associated with disturbed blood flow being either ischemia or bleeding. The presence of atherosclerotic lesions within arterial walls impairs cerebral blood flow and causes cerebrovascular dysfunction [109]. Most ischemic strokes are caused by atherosclerosis. According to a meta-analysis, the presence of carotid atherosclerosis was associated with an increased risk of recurrent stroke (OR: 2.87; 95%CI (2.42–3.37); *p* < 0.00001) [110]. Acute ischemic stroke leads to a critical limitation of blood supply, which results in neuronal cell death and cognitive decline. Cognitive impairment affects 20–80% of patients after acute brain ischemia [111]. Although SGLT2 inhibitors do not reduce the risk of ischemic stroke incidence, they affect the most important cerebrovascular risk factors, including hyperglycemia, hypertension, obesity, dyslipidemia, and atherosclerosis [112]. Hypertension is the most common risk factor of stroke [113]. SGLT2 inhibitors significantly lower systolic and diastolic blood pressure without reflex activation of the sympathetic nervous system and are even able to change the non-dipping to dipping circadian blood pressure profile. While the exact mechanism of the antihypertensive effect of SGLT2 inhibitors has not been clearly established, it is considered that the most important factors are osmotic diuresis (induced by glucosuria) and natriuresis. Other features of SGLT2 inhibitors that contribute to lowering blood pressure are suppression of the renin-angiotensin system, decreased activity of the sympathetic system, antioxidative activity, and improvement in endothelial cell function [114]. Moreover, SGLT2 inhibitors may improve brain damage and cognitive impairment in patients after a stroke. SGLT receptors are important in ischemia-reperfusion cerebral damage. As presented in a mouse model of subcortical white matter infarct with cognitive impairment, the knockout of the SGLT1 receptor was associated with a lower expression of proinflammatory cytokines and better cognitive performance [115]. SGLT1 receptors mediate sodium influx, which causes depolarization and contributes to neuronal cell death during ischemia. According to Yamazaki Y. et al., increased sodium influx via the SGLT1 receptor was associated with more exacerbated neuronal damage, which was not observed in SGLT-1 knockdown mice (Figure 4) [116]. In a study assessing cerebral ischemia/reperfusion damage in a rat model, empagliflozin, in a dose-dependant manner, reduced neuronal death, infarct size and ameliorated cognitive impairment via HIF-1α/VEGF signaling [50]. SGLT2 inhibitors may preserve cognitive functions in diabetic patients by preventing neurovascular remodeling and reducing the well-known risk factors of stroke. They can also bring benefits to post-stroke patients by reducing inflammation, sodium influx, and HIF-1α/VEGF pathway.

## 8. The Effect of SGLT2i on Alzheimer’s Disease Pathology

SGLT2 inhibitors can possibly bring benefits in patients with Alzheimer’s Disease using the abovementioned mechanisms, including not only anti-inflammatory, anti-oxidative or atheroprotective effects, but also direct neuroprotective effects including BDNF increase and AChE inhibition. Additionally, SGLT2i can also be favorable for AD patients by improving brain insulin sensitivity [100]. Insulin resistance is present in 8 out of 10 patients suffering from Alzheimer’s Disease [117]. Peripheral resistance to insulin also occurs in the CNS, as the glucose metabolic rate is reduced in the brains of AD patients in fluorodeoxyglucose positron emission tomography (FDG PET) [118,119]. Increased insulin level in the brain contributes to Alzheimer’s Disease pathology, as the insulin-degrading enzyme (IDE) also takes part in degrading senile plaques, and in insulin resistance, it is involved in degrading insulin [117]. Moreover, insulin resistance is associated with activating GSK3-β (glycogen synthase kinase 3β) signaling, which takes part in tau phosphorylation and Aβ production and Aβ mediated neuronal damage [117,120]. SGLT2 inhibition reduced GSK3-β activity in hepatocytes [121]. In previous studies involving murine models, SGLT2i treatment caused a significant reduction in AD pathology, including tau phosphorylation and senile plaques density. This effect was associated with the improvement in cognitive functions, including memory and learning processes in the new object discrimination test and Morris water maze test [15]. 

## 9. Summary

Type 2 Diabetes Mellitus, atherosclerosis, and cognitive impairment still remain global health problems as they are chronic, incurable diseases leading to a reduction in life quality and expectancy. All these diseases share many pathological pathways. In the era of tailored-made therapies and novel drugs with numerous pleiotropic effects, it is very important to seek for shared molecular pathways of commonly occurring diseases and redefine indications for commonly used medications since such solutions may be beneficial for patients. In this review, we have discussed the role of SGLT2 inhibitors used in diabetic patients for the prevention of atherosclerosis and cognitive impairment. Flozins may bring positive effects in T2DM, atherosclerosis, and cognitive impairment through several mechanisms, including anti-inflammatory and anti-atherosclerotic properties, SGLT1 inhibition, AChE inhibition, reduction in oxidative stress, amelioration cerebrovascular remodeling and restoring a balance between catabolism and anabolism. However, long-term clinical trials are necessary to establish whether the above-mentioned mechanisms are clinically relevant since atheroprotective and neuroprotective effects will not be immediate and require a long-term SGLT2i intake. Currently, the University of Kansas Medical Center (NCT03801642) is conducting a clinical trial on dapagliflozin in patients with Alzheimer’s disease.

## Figures and Tables

**Table 1:** Comparison of pleiotropic effects of Sotagliflozin, Canagliflozin, Dapagliflozin, Empagliflozin, Ertugliflozin.

	Sotagliflozin	Canagliflozin	Dapagliflozin	Empagliflozin	Ertugliflozin
SGLT2 Selectivity over SGLT1	20 fold [28]	250 fold [28]	1200 fold [28]	2500 fold [28]	2500 fold [28]
Brain/Serum Ratio	n/a	0.3	0.3	0.5	n/a
AChE Inhibition	*K*_i_ 5.6µM [29]	The most potent, even called a dual inhibitor *K*_i_ 0.13 µM [29]	*K*_i_ 25.02µM [29]	*K*_i_ 0.177µM [30]	*K*_i_ 31.69µM [29]
BDNF Increase	n/a	n/a	n/a	Yes [31]	n/a
Anti-epileptic Potential	n/a	n/a	Yes [32]	n/a	n/a
CIMT Regression	n/a	n/a	Yes [33]	Yes [34]	n/a
Anti-inflammatory	n/a	Yes [35]	Yes [36]	Yes [37]	No|n/a [38]
Blood-brain Barrier Protection	n/a	n/a	n/a	Yes [37]	n/a
NLRP3 Inflammasome Inhibition	n/a	n/a	Yes [39]	Yes [40]	n/a
Promoting M2 Macrophages Polarization	n/a	Yes [41]	Yes [42]	Yes [43]	n/a
Oxidative Stress Reduction	Yes [44]	Yes [45]	Yes [46]	Yes [47]	Yes [48]
Neurovascular Unit Remodeling	n/a	n/a	n/a	Yes [49]	n/a
Cerebral Ischemia/Reperfusion Damage Reduction	n/a	n/a	n/a	Yes [50]	n/a
Reduced mTOR Signaling	n/a	Yes [51]	Yes [51]	Yes [52]	Yes [53]
